# Family Medicine Program in Iran: SWOT Analysis and TOWS Matrix Model

**Published:** 2019-06

**Authors:** Hossein DARGAHI, Ali DARRUDI, Rostam ZALVAND

**Affiliations:** 1. Health Information Management Research Center, Tehran University of Medical Sciences, Tehran, Iran; 2. Department of Health Care Management, School of Public Health, Tehran University of Medical Sciences, Tehran, Iran

**Keywords:** Family medicine program, SWOT analysis, TOWS matrix strategy, Attending physicians, Iran

## Abstract

**Background:**

We aimed to determine strength, weakness, opportunities and threats analysis and intended to present strengths, weaknesses, opportunities and threats matrix model for appropriate implementation of Family medicine program in Iran.

**Methods:**

This was a descriptive-analytical and cross-sectional study. All attending physicians in 30 health care centers of Tehran University of Medical Sciences, Tehran, Iran were asked to present and prioritized their views about strengths, weaknesses, opportunities, and threats factors of family medicine program in Iran in 2015. Then, the prioritization of these factors was showed by weighted score of each factor. Finally, the respondents determined four groups of TOWS model including SO, ST, WO, and WT strategy for development of family medicine in Iran.

**Results:**

Totally, the respondents expressed 44 factors as strengths, weaknesses, opportunities, and threats of family medicine program and prioritized these factors and suggested 30 TOWS matrix strategy for efficient implementation of this program.

**Conclusion:**

There were many internal and external factors that impress the implementation of family medicine program. There is a gap between the ideal and the current situation of this program. We suggest the health care system policy makers notice the TOWS matrix strategies determined for improvement of family medicine program in Iran.

## Introduction

Comparisons of health care systems in all countries have shown a relationship between well-structured health care plan and family medicine because of lower total costs (1). The current structure of family medicine have developed in recent decades and have ability to change circumstances and important position in health care system (2). Evidence of health care system have been able to distinguish family medicine from the other aspects of health care delivery system (3). Several articles confirm success in health care system structure transformation including more effective care, more service oriented, more efficient care, and creating an effective environment (4–8). There are three main factors for better implementation of family medicine, including improvement of referral system, better availability to health care, and management of chronic disease improve the PHC in Iran (9–11).

However, family medicine program involves multiple problems in Iran not reflected adequately. Although, Lakarani “et al”. believed that financial resource deficit is the main challenge to family medicine program in Iran (12). Also, Takian (2009) revealed lack of cooperation, inappropriate environment and lack of sufficient experience of implementation are three challenges of family medicine program in Iran (13). Moreover, strong dissatisfaction among Iranian physicians because of high workload and inappropriate payment, lack of sufficient governmental support, replacement of managers and policymakers, little Gross Domestic Product (GDP) of the country, are the other challenges of family physician program in Iran (14–16).

It seems development of informative programs, more participation and involvement of physicians, the improvement of PHC network, customized framework of organizations, ideas, and believes may solve the difficulties of family medicine program in Iran (17–19). In the other words, family medicine program can be useful for improvement of preventive medicine, health care promotion, introduce the best services by the insurance organizations, health authorities and create any limitation of direct admission of patients by specialists and sub specialists (20–22).

According to SWOT analysis which identify their internal and external factors of organizations and where the TOWS matrix becomes a useful and effective tool to determine strategic planning helps to improve decision making in health care system (23–26). Two studies reported the results of SWOT analysis of family medicine plan in Iran and explained the strengths, weaknesses, opportunities and threats of this plan (27–28).

This research was aimed to determine SWOT analysis of family medicine program from the view of attending physicians worked in health care centers networks of south of Tehran affiliated to Tehran University of Medical Sciences as the largest and the best and more efficient health care network in Iran. Moreover, this research is intended to respond to these questions:

1- What are the weakness, strength, opportunities, and threats of the family medicine project,
2- What are the strategies of WT, ST, WO, and SO for improvement of this project?

## Methods

This descriptive-analytical study was induced cross-sectional in order to determine TUMS attending physicians’ view about SWOT analysis of family physician program at Iran in 2015. The study population of this research was 42 as all attending physicians in 30 health care centers of network south of Tehran, Iran. The sample size was purposefully selected and was equal to the study population. The research tool, the SWOT questionnaire, was constituted by the researchers using SID, Magiran, Iran Medex, Google, Google Scholar, Medline, Emerald, PubMed databases with keywords of Family Medicine Program, Physicians, Iran, SWOT analysis, and studied 115 references from 2000–2016, and selected 40 references related to this research. Collected information from these resources was categorized into 8 strengths, 15 weaknesses, 8 opportunities, and 9 threats and constituted as SWOT analysis questionnaire. Then the questionnaire was given to 15 experts, researchers, and policy makers of TUMS in the field of family medicine program to calculate Content Validity Index (CVI) as 0.85.

Regarding the reliability of the questionnaire and calculation of the internal coordination and stability of the items, Alpha Chronbach technique was employed which was 80%. Test-retest and Interclass Correlation Coefficient (ICC) methods were used to examine the repeatability of the questionnaire. To this end, the questionnaire was distributed among 15 non-participant physicians who were expert about family medicine program and were collected once completed. This questionnaire were again handed out to the physicians after 15 d intervals to avoid recalling error. After examining and analyzing the results of these two stages, interclass Correlation Coefficient of the questionnaire was 85%.

For selection and using TOWS model, and determination of Internal Factor Evaluation (IFE), and External Factor Evaluation (EFE), the researchers provided the questionnaire personally to 42 attending physicians and asked them to prioritize the strengths, weaknesses as internal factors, opportunities, and threats as external factors influenced the success of family medicine program in Iran. In order to obtain the weights of the factors, all of them were assigned with relationship importance rating from 1–10 to indicate the relative importance of family medicine program, prioritized by the attending physicians. The score of 10 indicated extremely important, and the score of 1 assigned the factor was extremely not important. Therefore, the scores from 1 to 10 showed the relative importance of each factor from extremely important to extremely not important from the views of the attending physicians. The average of numbers divided into the sum total numbers showed the “weight” calculation of the factors. In addition, this questionnaire assigned from 1 to 4 to indicated the prioritization of family medicine program by the attending physicians, while the score of 4 indicated that the factor was the main strength, and the score of 1 indicated that the factor was the main weakness which showed by “Average Score”. Finally, the product of “weigh” with “Average Score” that showed “weighted score” defined the priority of family medicine program factors in Tables 1 and 2.

**Table 1:** TOWS analysis matrix

***Internal factors External factors***	***Strengths***	***Weaknesses***
Opportunities	SO Strategy (Area 1) Maximization of opportunities by application of strengths (SO)	WO Strategy (Area 2) Using potentially benefits hidden into opportunities for compensation of weaknesses
Threats	ST Strategy (Area 3) Using strengths for prevention of exposure to threats (ST)	WT Strategy (Area 4) Minimization of loss of threats and weaknesses

**Table 2:** Strengths and weaknesses family medicine program from view of attending physicians of TOWS

***Strengths***	***Weight***	***Average Score***	***Weighted Score***	***Priority***

Easy access villagers to physicians	0.033	3.58	0.118	1
Annual checkups and household filing	0.031	3.46	0.108	2
More effective care delivery for mothers and children under six years	0.032	3.33	0.106	3
Reducing the cost of patients’ therapy	0.030	3.38	0.103	4
Improving access to physicians and drugs	0.030	3.17	0.095	5
Patient screening and early detection of diseases	0.029	3.04	0.089	6
Promoting community health indicators	0.028	2.92	0.082	7
Encouraging health workers and health homes instructors	0.018	1.67	0.030	8
***Weaknesses***	***Weight***	***Average Score***	***Weighted Score***	***Priority***
Increasing the workload of physicians, midwiferies and health workers	0.036	3.54	0.127	1
Limited access to family physicians	0.035	3.33	0.115	2
Crowding of clients to health homes	0.033	3.42	0.113	3
Being problem in the patient’s hospital admission	0.032	3.33	0.106	4
Lack of job security for physicians	0.026	2.83	0.075	5
Insufficient orientation of physicians and specialists	0.027	2.75	0.074	6
Lack of timely payment of salaries for physicians and midwives	0.024	2.79	0.066	7
Proving inadequate training to physicians about health problems	0.026	2.54	0.065	8
Lack of integrated management	0.023	2.38	0.055	9
Lack of selection of family physician by the patient	0.020	2.63	0.054	10
Lack of appropriate allocation amenities	0.018	2.58	0.046	11
Inefficient of information systems in hospitals and health centers	0.019	2.46	0.046	12
Lack of orientation of villagers to the family medicine program	0.021	2.17	0.045	13
Persistent of physicians, nurses, midwives and health workers versus health care change plan	0.016	2.33	0.037	14
Poor deal of villagers with medical staff	0.015	2.25	0.033	15

This study was approved by the Ethical Research Committee in Tehran University of Medical Sciences. Responding to the questionnaire was voluntary, and all answers were re-identified to maintain confident ability and placed into the researchers’ close closet.

The data was analyzed by SPSS (Chicago, IL, USA), and descriptive statistics presented by average score, weighted score, and priority. Consequently, the authors designed TOWS analysis matrix for determination of four groups of strategies as TOWS model (Table 1).

## Results

Overall, 32 internal and external factors impressed the success of family medicine in Iran from the attending physicians’ views of Tehran University of Medical Sciences. There were 8 strengths and 15 weakness factors that influenced the family medicine program as Internal Factor Evaluation (IFE). “Easy access villages to physicians” with average weight score of 3.58 is the first priority, and “Encouraging health workers and health home instructors” with average weight score of 1.67 was the last priority of strengths factors as IFE impress the family physician program in Iran. Moreover, “increasing the workload of physicians, midwiferies, and health workers”, was the first, and “poor deal of villages with medical staff” was the last priority of weaknesses factors influence the family medicine program in Iran, retrospectively (Table 2). There were 17 external factors, including 8 factors related to opportunities and 9 factors related to treats influence as the External Factor Evaluation (EFE) matrix of family medicine program.

The highest priorities of opportunities were “reduce the cost of treatment in health care system” with average weight score of 3.83. Moreover, the highest priorities of treats were “lack of financial supporting by the government”, and “inadequacy of per capita health” with average weight score of 3.50 and 3.54 (Table 3).

**Table 3:** Opportunities and threats family medicine program from view of attending physicians of TUMS

***Opportunities***	***Weight***	***Average Score***	***Weighted Score***	***Priority***

Reduce the cost of treatment in the health system	0.032	3.83	0.124	1
Distribution of public insurance	0.034	3.63	0.123	2
Create job opening for general physician	0.033	3.38	0.110	3
Establishment of reasonable patterns in the uses of drugs and medical equipment	0.024	2.88	0.069	4
Existence of teamwork views in health care system	0.016	2.13	0.035	5
Ensuring equity in health system	0.016	1.92	0.030	6
Relieve the concerns of senior officials based on resolve all the problems of patients except for suffering from diseases	0.014	1.79	0.026	7
Availability of specialized physician in all fields, including consultation, treatment, and decision-making for patient	0.016	1.67	0.026	8
***Threats***	***Weight***	***Average Score***	***Weighted Score***	***Priority***
Lack of financial supported by the government	0.034	3.50	0.118	1
Inadequacy of per capita health	0.029	3.54	0.101	2
Improper cooperation from specialists	0.027	3.08	0.083	3
Unstable state for compensation service of physicians	0.024	2.63	0.062	4
Lack of appropriate structure of communication	0.020	2.54	0.052	5
Change of policies because of change of governments	0.021	2.58	0.055	6
Low intersectional coordination	0.019	2.54	0.049	7
Gap between the levels of compensation among physicians, nurses, midwives and health workers	0.021	2.21	0.047	8
Desecend quality in health care	0.018	2.29	0.042	9

Although “availability of specialized physicians in all fields with average of score of 1.67 as the lowest opportunities factor, and “Descend quality in care” with average weight score of 2.21 as threats factor was the lowest priorities of external factors influence the family medicine program from the attending physicians’ views of Tehran University of Medical sciences. Table 3 showed the summary selected views of the physicians in order to support family medicine implementation.

Seven W-T strategies minimize weaknesses in order to reduce threats of family medicine program. In addition, there are 5 S-T strategies use strengths to minimize the effect of threats of family medicine program (Table 4).

**Table 4:** TOWS strategies for promotion of family medicine program in Iran

***(W-T) Strategies***	***(S-T) Strategies***

1. Improve the capacity of health workers and midwives to provide basic and simple services to patients in order to prevent loss of human resource and a reduction in state payments 2. Make continuing evolution of services system 3. Use of voluntary service 4. Forecast and paying special bonuse to employees of the sector and the induction of effective and efficient encourage system 5. Use of new technology in adoption and follow-up 6. Recovery and renovation and beautification of old buildings 7. Reduce the covered population of physicians	1. Participation of private sector to invest in the area of health care 2. Attract physicians of health system to support the University of medical sciences 3. Creating mechanisms for public participation in the supply of health care 4. Support for tariff increases of general practitioners in health centers 5. Carry out systematic and rational decision-making in health care
***(W-O) Strategies***	***(S-O) Strategies***
1. Creating job opportunities for general physicians 2. Efficient training for physicians and health workers in order to reduce treatment cost 3. Establishment of research & development 4. Selection and application of efficient staff 5. Increased of organizational commitment 6. Establishment of change management 7. Establishment of interactive leadership system	1. Development of public insurance in order to reduce to reduce paying from people’s pocket 2. Easy access of villagers and failed people to physicians and drugs 3. Establishment of rational patterns for drugs consumption and using the instrument 4. Patient findings and fast diagnosis of diseases 5. Priority of prevention 6. Appropriate communication and interaction between people with society health 7. Positive view of people about health care system 8. Increased empowerment and efficiency of health care system 9. Corresponding influences of people’s satisfaction about political decision-making

Moreover, Table 3 shows 7 W-O strategies for compensation of weaknesses through opportunities of family medicine or minimize weaknesses by taking advantages of opportunities. Although, S-O strategy apply strengths for maximization of opportunities of family medicine program. After prioritizing of internal and external factors influenced the family medicine program in Iran by attending physician of Tehran University of Medical Sciences, and weighted score of each factor, they were asked to present their comments about the TOWS model for development of family medicine program in Iran.

## Discussion

Today’s world clearly accepted the rights of the people to have the best performance of health care system (27). Family medicine physicians take responsibility and commitment to primary health care that usually are the first invitation in case of illnesses or accidents and consulting services and treatment (28).

Furthermore, SWOT analysis could not determine whether the situations “good” or “bad”. It is a procedure used to predict the different ways at a given moment (29). SWOT analysis shows that there are clear strengths, weaknesses, opportunities, and threats in the implementation of family medicine program in Iran (30). Strategic planning as a reflex of values identifies the limitations of internal and external of organizations (29). SWOT analysis and determination of WT, SO, WO, and ST strategies are based on creativity and select views in order to support for preparation of this strategy (31).

This research is the first study to determine SWOT analysis of family medicine program from the views of attending physicians worked in Tehran University of Medical Sciences (TUMS) as the largest and the best of medical university in Iran. Moreover, TUMS has the highest international scientific ranking in the world in comparison with the other Iranian universities. Therefore, the results of this research could generalize into all of Iran.

In our study, the attending physicians believed there were several strengths and weaknesses as internal evaluation of family physician program in Iran. Availability to the physicians and drugs, effective care of mothers and children’s, promoting health care indicators, reduction of cost and promotion of health workers are the higher priorities strengths of family physician from attending physicians’ views. This is compatible with the study induced in North Western of Iran (30). In addition, the patients were satisfied with communication skills of family physicians in Jahrom, Fars Province in Iran (32). The most rate of patient satisfaction from family physician program was the cost reduction of health care delivery in Northern Eastern of Iran (33). Our study results about the strengths factors of family medicine program were confirmed by other studies (34–36).

In current study, attending physicians of TUMS believed that increasing of medical practitioners’ workload, being problem in the referral system, inefficient orientation of physicians, lack of integrated management, and inefficient of information systems were the prioritized weaknesses of family physicians program. Many regulations of referral system were not observed as a feedback for family medicine program in Northern Province of Iran (31) that is compatible with current study results. Although, another study confirmed this section of our study results (37). Inappropriate of referral system, insufficient electronic health record, lack of orientation of physicians were the weaknesses of family physician program from Kashan University of Medical Sciences physicians’ view in Iran (38).

Existence of teamwork view in health care system, availability of specialized physician in all fields, ensuring equity in health care system, and creating job opening for general physicians were the lowest opportunities, and lack of financial support, inappropriate of communication, low intersectional coordination were the higher prioritized threats of family physician program in Iran from views of TUMS attending physicians. Integration and coordination of PHC and family medicine program are essential (9). Financial problem is the main challenge of family medicine program in Iran (10). All challenges of family medicine program were summarized into inappropriate environment and lack of experiences to implement this program (12) that are compatible with the threats factors of family medicine program in our study.

Moreover, another study confirmed the threats of family medicine program expressed in current study (13). The conflict of interest of different policy makers of Iranian health system to integrate between family medicine program and referral system (15) that is similar to current study results. Although, the opportunities of family medicine program in the other countries are not similar to those presented in Iran (17, 18). The result of SWOT analysis of family medicine in Iran (24) was not similar to our findings. This difference is related to research society and different views of respondents.

Many organizations utilize SWOT analysis to identify their internal and external factors that impress their performances (24). The resulting information of SWOT analysis is where the TOWS matrix becomes a useful tool. TOWS matrix model is a simple and effective tool to determine the specific strategic planning for attaining the SWOT investigation (25). TOWS matrix are a strategic planning helps to improve decision making in health care system (26). In current study, for the first time we could determine TOWS matrix as useful tool by brainstorming of attending physicians of TUMS to present effective strategies for policy makers of Iranian health care system.

The most remarkable limitation of the present research is due to the fact that all attending physicians prioritized the strengths, weaknesses, opportunities and threats of family medicine program in Iran based on self-report to obtain the weighs of the IFE and EFE factors and prioritized the main strength and weakness to show the average score of family medicine program factors.

## Conclusion

Family medicine program is a vital tool for essential reforms in the current challenges of Iranian healthcare system. Implementation of family medicine program is a powerful mean to strengthen the PHC model in rural-urban areas. There is a gap between the ideal and the current situation of family medicine program.

There were many internal and external factors that impress the implementation of family medicine program in Iran. These factors including strengths, weaknesses, opportunities, and threats prioritized by expert specialist physicians and induced the weighted score of each factor impress the family medicine program. The policy makers of Iranian health care system notice the TOWS matrix determined in this article for successful implementation of family medicine program.

## Ethical considerations

Ethical issues (Including plagiarism, informed consent, misconduct, data fabrication and/or falsification, double publication and/or submission, redundancy, etc.) have been completely observed by the authors.
